# Alkaloid and benzopyran compounds of *Melicope latifolia* fruit exhibit anti-hepatitis C virus activities

**DOI:** 10.1186/s12906-021-03202-8

**Published:** 2021-01-12

**Authors:** Aty Widyawaruyanti, Mulyadi Tanjung, Adita Ayu Permanasari, Ratih Saputri, Lidya Tumewu, Myrna Adianti, Chie Aoki-Utsubo, Hak Hotta, Achmad Fuad Hafid, Tutik Sri Wahyuni

**Affiliations:** 1 Department of Pharmacognosy and Phytochemistry, Faculty of Pharmacy, Universitas Airlangga, Jl. Mulyorejo, Surabaya, 60115 Indonesia; 2 Institute of Tropical Disease, Universitas Airlangga, Jl. Mulyorejo, Surabaya, 60115 Indonesia; 3 Department of Chemistry, Faculty of Science and Technology, Universitas Airlangga, Jl. Mulyorejo, Surabaya, 60115 Indonesia; 4 Department of Public Health, Kobe University Graduate School of Health Sciences, 7-10-2, Tomogaoka, Suma-ku, Kobe, 654-0142 Japan; 5 Faculty of Clinical Nutrition and Dietetics, Konan Women’s University, 6-2-23 Morikita-machi, Higashinada-ku, Kobe, 658-0001 Japan

**Keywords:** *Melicope latifolia*, Hepatitis C virus, Alkaloids, Benzopyrans

## Abstract

**Background:**

New agents for developing alternative or complementary medicine to treat the hepatitis C virus (HCV) are still needed due to high rates of HCV infection globally and the current limitations of available treatments. Treatment of HCV with a combination of direct acting antivirals have been shown to be approximately 90% effective but will be limited in the future due to the emergence of drug resistance and high cost. The leaves of *Melicope latifolia* have previously been reported to have anti-HCV activity and are a potential source of bioactive compounds for future novel drug development. This study aimed to evaluate the efficacy of the extract of *M. latifolia* fruit to treat HCV and to isolate its active compounds.

**Method:**

*M. latifolia* fruit was extracted using methanol and purified using vacuum liquid chromatography (VLC) and Radial Chromatography. The anti-HCV activity was analyzed using cell culture lines Huh7it-1 and JFH1 (genotype 2a). Time-of-addition and immunoblotting studies were performed to identify the mode of action of the isolated active compounds. The structures of the active compounds were determined using nuclear magnetic resonance (NMR) spectra, UV, IR, and Mass Spectra.

**Results:**

Six known compounds were isolated from *M. latifolia* fruit: O-methyloktadrenolon, alloevodionol, isopimpinellin, alloxanthoxyletin, methylevodionol, and N-methylflindersine. N-methylflidersine was the most active compound with IC_50_ value of 3.8 μg/ml while methylevodionol, isopimpinellin, and alloevodionol were less active. O-methyloktadrenolon and alloxanthoxyletin were moderately active with IC_50_ values of 10.9 and 21.72 μg/ml, respectively. N-methylflidersine decreased level of HCV NS3 protein expression in the cells.

**Conclusion:**

The alkaloid compound, N-methylflindersine which was isolated from *M. latifolia* possesses anti-HCV activity through post-entry inhibition and suppressed NS3 protein expression.

## Background

The hepatitis C virus (HCV) is a pathogen that causes liver inflammation that can develop into cirrhosis and or hepatocellular carcinoma. No vaccine is presently available against HCV due to their extreme genetic variability and lack of animal models for vaccine testing [1, 2]. Seven genotypes of HCV are known to exist and are transmitted by sexual contact, sharing of injection equipment, reuse or inadequate sterilization of medical equipment and non-screened blood transfusion [2].

The current treatment of HCV uses direct acting antivirals (DAAs) which increased sustained virologic response (SVR) > 90% of people in clinical trials. DAAs treatment is divided into four classes: protease inhibitors, non-nucleoside polymerase inhibitors, nucleoside/tide polymerase inhibitor, and NS5A inhibitors [3–5]. However, drug resistance and the affordability of treatment access in low income countries prevent their widespread use [5, 6]. Potentially, new antiviral drugs may be able to be developed from plants with low side effects as there have been numerous reports on the antiviral activity of various phytochemicals against HCV [7–9].

Medicinal plants contain diverse chemical compounds that possess many potential bioactivities including anti-HCV properties. Isolated compounds from a diverse range of medicinal plants have been reported as having anti-HCV properties such as saikosaponin b2, α-mangostin, oleanolic acid, ursolic acid, EGCG, glycyrrhizin, chalepin, and pseudane IX [7–10]. Chalepin and pseudane IX, isolated from *Ruta angustifolia*, exhibited anti-HCV activity by reducing the virus particle production and decreasing HCV NS3 protein level [11]. Some active compounds from plants have been so successful that they have reached clinical trials such as naringenin and silymarin/silibinin [12].

*M. latifolia* belongs to *Rutaceae* family. The leaves of *M. latifolia* were used as traditional medicine for the treatment of fever, cramps, jaundice and malaria [13]. Several species of *Melicope* are reported to possess flavonoids, alkaloids, coumarins and other metabolites [14, 15]. These compounds possess potential bioactivities and have previously been shown to have anti-viral activity [14]. The alkaloid, pseudane IX and a benzopyran compound, chalepin, isolated from *R. angustifolia* have exhibited anti-HCV activities. The alkaloid compound, namely APS, isolated from *Maytenus ilicifolia*, also exhibited anti-HCV activity by inhibited HCV replication and significantly reduced HCV NS5A [8]. Two novel myrioneuron alkaloids, schoberine B and myriberine B were isolated from aerial part of *Myrioneuron faberi* [16]. These alkaloids compounds were evaluated against HCV and showed strong anti-HCV activity. Various compounds from many of genus *Melicope* have been isolated. The leaves of *M. triphylla* are reported to contain 5,8-dihydroxy-3,7dimethoxy-3,4-methylenedioxyflavone, 7-hydroxy-3,5-di-methoxy-3′,4′-methylenedioxyflavone, 7-(2,3-dihydroxy-3-methylbutoxy)-3, 5-mimethoxy-3′,4′-methylene-dioxyflavone, 7-(2,3-dihydroxy-3-methylbutoxy)-3–3′,4′,5-tetramethoxyflavone, and 7-(2,3-dihydroxy-3-methylbutoxy)-3,3′4’,5,8-pentamethoxyflavone. The O-prenylated flavonoid (3,5,4′-trihydroxy-8,3′-dimethoxy-7-(3-methylbut-2-enoxy) flavone was isolated from *M. pteleifolia* [14]. Two quinoline alkaloids, buchapine and 3-(3-methyl-2-butenyl)-4-[(3-methyl-2-butenyl)oxy]-2(1H)-quinolinone, and three furoquinoline alkaloids, roxiamines A, B, and C were isolated from flowers, leaves, and twigs of *M. lunu-ankenda* and the quinoline alkaloids revealed anti-HIV activity [17]. Our previous screening of 21 plants from Indonesia revealed that the ethanol extract of *M. latifolia* leaves exhibited anti-HCV activity with an IC_50_ of 3.5 and 2.1 μg/ml against HCV J6/JFH1-P47 and -P1 strains respectively. The cytotoxicity concentration 50% (CC_50_) was found to be > 100 μg/ml. The mode of action of the tested extracts was through their action in the entry and post-entry steps of HCV life cycle. Moreover, the HCV NS3 protein level has also been reported to be suppressed by the *M. latifolia* extract [10]. The fraction of the *M. latifolia* leaf extract which contained the alkaloid compound, N-methylflindersine, showed a strong inhibition against HCV [15]. These results may provide a potential to find anti-HCV compounds from other parts of *Melicope latifolia*, Therefore, this current study was conducted to determine the anti-HCV activity of the *M. latifolia* fruit. We then further fractioned, purified and isolated of its active constituents to obtain anti-HCV compounds.

## Methods

### Cells and viruses

Hepatocyte cells of Huh7it-1 [18] were cultivated in DMEM-Dulbeco’s Modiffied Eagle Medium (GIBCO Invitrogen). The medium was supplemented with 10% Fetal Bovine Serum (FBS, GIBCO-Invitrogen), 0.15 mg/ml kanamycin solution (SIGMA) and 1x Non-Essential Amino Acids (NEAA, GIBCO-Invitrogen). The cell culture was incubated in 5% CO_2_ at 37 °C. HCV JFH1a was propagated in Huh7it-1 suspended in 4 ml JFH1 suspension and 4 ml DMEM and was incubated at 37 °C in 5% CO_2_ for 4 h. The suspension was divided into eight flasks and the supernatant was harvested on the third day. The supernatant was concentrated and examined for its viral titration and stored at − 80 °C [10].

### Extraction and isolation of benzopyran compounds of *Melicope latifolia* fruit

*M. latifolia* were collected at Cangar forest, East Java, Indonesia. The collected plants were verified by botanist researchers at Purwadadi Botanical Garden-Indonesia Institute of Science, Purwadadi, East Java, Indonesia (Number of determination: 0340/IPH.06/H/III/2017). The voucher specimen has been deposited in Institute of Tropical Disease, Universitas Airlangga. The fruits of plant were dried at room temperature and pulverized. Dried and powdered fruits of *M. latifolia* (450 g) were extracted using methanol as a solvent and yielded 25.5 g of dried methanol extract. The extract was further partitioned using n-hexane. The dried n-hexane extract (14.0 g) was then subjected to vacuum liquid chromatography (VLC) using silica gel as the stationary phase and n-hexane:ethyl acetate in a gradient composition (9:1; 8.5:1.5; 8:2; 1:1) as the mobile phase. Five fractions (Fraction A-E) were obtained. Among the fractions, fraction B was observed as intense violet spots under UV light which indicated the presence of coumarin compounds [19]. Further separation of Fraction B (7 g) was conducted by VLC using n-hexane:ethyl acetate in a gradient composition (9.5:0.5 until 8:2) as the mobile phase and yielded six sub-fractions (B1-B6). Separation of B3 (3 g) by radial chromatography using n-hexane:diisoprophyl ether in a gradient composition (9.5:0.5 until 8:2) yielded three further sub-fractions (B31, B32 and B33). Purification of B31 (49.5 mg) by radial chromatography using n-hexane:diisoprophyl ether in a gradient composition (9.75:0.25 until 8:2) yielded a benzopyran compound (O-methyloktadrenolon; 26.8 mg) in the form of a pale yellow oil. Purification of B32 (107.9 mg) by same method, yielded a benzopyran compound (alloevodionol; 49.8 mg) in form of a pale yellow solids.

### Extraction and isolation of alkaloid compounds of *Melicope latifolia* fruit

The dried fruit of *M. latifolia* (500 g) was extracted using methanol as a solvent and yielded 115 g of concentrated methanol extract. The extract was further partitioned using n-hexane with a solvent composition of 1:1. The methanol phase was then separated and treated with citric acid until pH 3–4. Further partition of methanol phase was then conducted using ethyl acetate in which the ethyl acetate extract phase and alkaloid extract phase were obtained. The ethyl acetate extract phase was then dried to obtain the final ethyl acetate extract (33 g). Meanwhile, alkaloid extract phase was again partitioned using ethyl acetate and washed with distilled water until pH 7 to obtain alkaloid extract (5 g).

Separation of the ethyl acetate extract (33 g) was done by VLC using n-hexane: ethyl acetate as the solvent with increased polarity (9:1 until 3:7). Three main fractions were obtained (Fraction A-C). Fraction B and C were observed as intense violet spots under UV light. Fraction B (975 mg) was subjected to VLC using n-hexane:ethyl acetate (9:1 until 6:4) and chloroform:ethyl acetate (7:3 until 1:1) to obtain three sub-fractions (B1-B3). The Thin Layer Chromatography (TLC) analysis of B3 showed intense violet spots under UV light which indicated the presence of a coumarin compound [19]. Purification of B3 (105 mg) by radial chromatography using n-hexane:acetone (9.5:0.5 until 8:2) yielded a coumarin compound (alloxanthoxyletin: 12.2 mg) in the form of a white solid.

Separation of fraction C (625.0 mg) by VLC using n-hexane:ethyl acetate (0:1 until 7.5:2.5) yielded two sub-fractions, C1 and C2. Further purification of C2 (89.0 mg) by radial chromatography using n-hexane:chloroform (9:1 until 7:3) yielded a coumarin compound (isopimpinellin; 8.4 mg) in the form of a pale yellow solid.

Separation of fraction A (325 mg) by radial chromatography using n-hexane:chloroform (7:3; 1:1) and chloroform yielded three sub-fractions, A1-A3. Purification of A1 (141 mg) was done by radial chromatography using n-hexane:chloroform (9:1; 8:2; 7:3) to obtain benzopyran compound (methylevodionol; 41 mg) in the form of an oil.

The separation of the alkaloid extract was conducted by VLC to obtain three fractions (fraction A-C). The separation of Fraction A by radial chromatography using n-hexane:ethyl acetate (9:1; 8:2) yielded three sub-fractions (A1-A3). Radial chromatography was applied to purify A3 using n-hexane:acetone (9:1; 7:3) and obtained an alkaloid compound (N-methylflindersine; 12.3 mg) in the form of a yellow oil.

### Antiviral activity assay

The hepatocyte cells of Huh7it-1 were seeded on a 48 well plate with a cell density of 5.4 × 10^4^ and incubated for 24 h. Samples with concentrations of 100, 30, 10, 1, 0.1 and 0.01 μg/ml were mixed with HCV JFH1a with multiple infections (MOI 0.1) and then inoculated onto cells and incubated for 2 h. The supernatant was removed and washed with medium and placed back into solutions containing the same concentrations of extract. The cultures were then incubated for 48 h and the supernatant was taken to examine the titer infection of the sample by immunostaining [10].

### Time-of-addition experiment

A time addition experiment was performed to analyze the effect of the extract on the HCV life cycle. Entry inhibition was conducted by treated cells with extracts during viral inoculation to evaluate the inhibition in binding, entry and endocytosis. Post entry inhibition was conducted by treating cells with the extracts after viral inoculation. Supernatants were collected and viral titration and immunostaining was used to assess the mode of action [20].

### Viral titration and immunostaining

Viral titration was performed by serially diluting the culture supernatant in DMEM medium and inoculating onto Huh7it-1 cells. After incubation for 41 h, the cells were fixed by 3.7% formaldehyde and permeabilized with 0.5% triton X-100 for 10 min. First antibody (HCV infected serum patient) was used in 1% bovine serum albumin (BSA) /2%BlockIce/ phosphate buffer saline (PBS) with a 300x dilution then incubated for 60 min. Second antibody (HRP-Goat anti human) was used in 1%BSA/2%BlockIce/PBS with a 400x dilution (50 μl/well) and then incubated for 60 min at room temperature. To visualize the infectious foci, 3,3′-diaminobenzidine (DAB) staining (DAB Thermo Scientific, USA) was used for 15 min until a brown color was observed. SPSS probit was used to calculate inhibition concentration 50% (IC_50_) values.

### Cytotoxicity assay

The cytotoxicity analysis was assessed using MTT assay 3-(4,5-Dimethylthiazol-2-Yl)-2,5-Diphenyltetrazolium Bromide [20, 21]. Solutions with extract concentrations of 1000, 400, 100, 50, 10, 1and 0.1 μl were added onto Huh7it-1 cells in 96 well plates. After 46 h incubation, the medium was removed and 150 μl/well of 10% MTT was added then incubated for 4 h. The purple precipitates were then dissolved in dimethyl sulfoxide (DMSO). The purple solution was measured for UV absorbance using a microplate reader at 560 nm. SPSS probit was used to calculate the cytotoxic concentration 50% (CC_50_).

### Immunoblotting

Huh7it-1 cells were lysed with Ripa buffer and the amount of protein was checked using a bichincronic acid (BCA) assay kit. The proteins were separated in (sodium dodecyl sulfate polyacrylamide gel electrophoresis (SDS-PAGE) gel and transferred onto a PVDF membrane (Millipore, Bed-ford, MA, USA). Five percent skimmed milk was added to block non-specific binding and incubated for 60 min. First antibody, HCV NS3-specific mouse monoclonal antibody (clone H23; Abcam, Cambridge, MA, USA) and glyceraldehyde-3-phosphate dehydrogenase (GAPDH) antibody (MBL) was added and incubated for 1 h. The 0.05% PBST was used for membrane washing. Second antibody, HRP-conjugated goat anti mouse immunoglobulin (MBL) was incubated for 1 h and the respective protein was visualized using Clarity Western ECL substrate (Biorad). The chemical luminescence was detected using ImageQuant LAS 4000 (GE healthcare) [20].

## Results

### Identification of isolated compounds of *Melicope latifolia* fruit extract

**Fig. 1:**
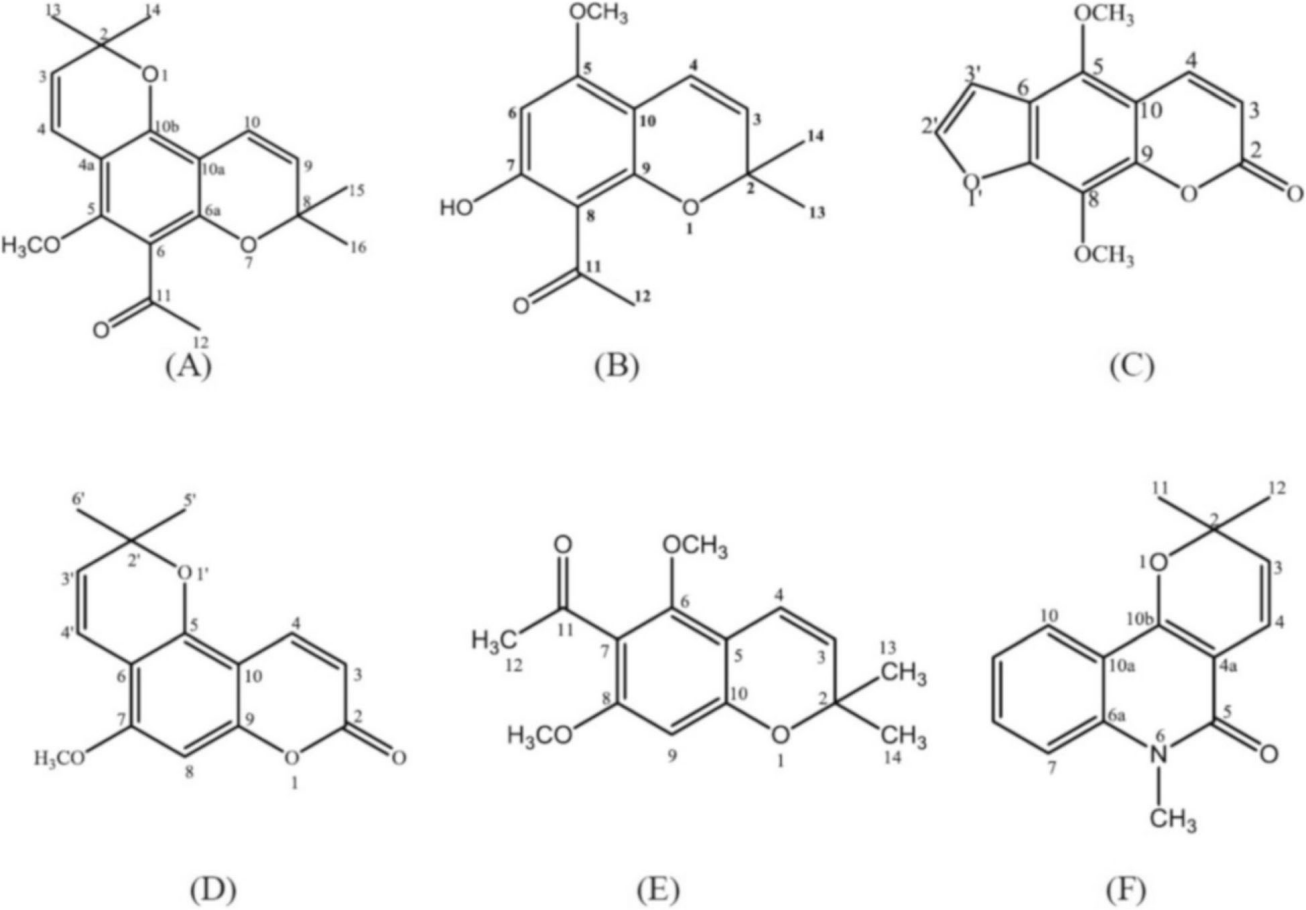
Chemical structure of isolated compounds of *Melicope latifolia* fruit. **a** O-methyloktadrenolon; **b** Alloevodionol; **c** Isopimpinellin; **d** Alloxanthoxyletin; **e** Methylevodionol; and **f** N-methylflindersine

Six known compounds were isolated from the *M. latifolia* fruit extract. Structure elucidation of compounds was determined by Nuclear Magnetic Resonance (NMR) spectra, ultra violet (UV), infrared (IR) spectroscopy and mass spectra (MS) data. NMR spectra were measured on a JEOL JNM-ECA 400 MHz FTNMR spectrophotometer in CDCl_3_ solvent. UV spectra were recorded in methanol on a Shimadzu series 1800 UV-VIS spectrophotometer. IR spectra were recorded in KBr on a One Perkin Elmer instrument. Mass spectra were measured on an ESI-TOF Waters LCT Premier XE producing pseudo-molecular ions, [M + H]^+^ positive ion mode. The molecular structures of isolated compounds are described in Fig. 1.

#### O-methyloktadrenolon

The compound showed a UV maximum absorption (MeOH) at λ_max_ nm (log ε): 217 (2.95); 223 (2.80); 278 (3.12); 289 (3.05) and 355 (2.14). IR spectrum showed a band at ν_max_ (cm^− 1^): 3055; 2974; 2935; 1697; 1639–1579; and 1184. The mass spectrum of the compound showed a quasi-molecular ion [M + H]^+^ at *m/z* 315.1595 corresponding to the molecular formula C_19_H_23_O_4._

^1^H-NMR (CDCl_3_) δ_H_ ppm: 5.51 (1H, d, *J* = 9.8 Hz, H-3), 6.48 (1H, d, *J* = 9.8 Hz, H-4), 5.53 (1H, d, *J* = 9.8 Hz, H-9), 6.59 (1H, d *J* = 9.8 Hz, H-10), 2.50 (3H, s, H-12), 1.42 (3H, s, H-13/14), 1.41 (3H, s, H-15/16), 3.75 (3H, s, 5-OCH_3_). ^13^C-NMR (CDCl_3_) δ_C_ ppm: 201.1 (C-11), 153.7 (C-5), 151.5 (C-6a), 150.6 (C-10b), 127.9 (C-3), 127.8 (C-9), 116.8 (C-10), 116.2 (C-4), 117.7 (C-6), 108.2 (C-4a), 106.6 (C-10a), 77.2 (C-8), 76.9 (C-2), 32.7 (C-12), 28.0 (C-13/14), 27.9 (C-15/16), 63.7 (5-OCH_3_). The NMR spectra data are consistent with published data [22].

#### Alloevodionol

The compound showed a UV maximum absorption (MeOH) at λ_max_ nm 217 (3.04) and 257 (3.24). IR spectrum showed a band at ν_max_ (cm^− 1^): 3051; 2970; 2864; 1639; 1610–1587 and 1205. The molecular formula was determined to be C_14_H_16_O_4_.

^1^H-NMR (CDCl_3_) δ_H_ ppm: 5.41 (1H, d, *J* = 10.1 Hz, H-3), 6.55 (1H, d, *J* = 10.1 Hz, H-4), 6.00 (1H, s, H-6), 2.66 (3H, s, H-12), 1.48 (3H, s, H-13/14), 13.83 (1H, s, 7-OH), 3.83 (3H, s, 5-OCH_3_). ^13^C-NMR (CDCl_3_) δ_C_ ppm: 203.4 (C-11), 166.4 (C-7), 161.0 (C-5), 156.3 (C-9), 124.7 (C-3), 116.6 (C-4),106.0 (C-8), 102.8 (C-10), 92.3 (C-6),78.0 (C-2), 33.2 (C-12), 27.9 (C-13/14), 55.8 (5-OCH_3_). The NMR spectra data are consistent with published data [22].

#### Isopimpinellin

The compound showed a UV maximum absorption (MeOH) at λ_max_ nm (log ε): 223.10 (4.20); 241.10 (4.00); 248.60 (4.00); 268.60 (4.09) and 311.30 (3.91). IR spectrum showed a band at ν_max_ (cm^− 1^): 3128; 2952; 2850, 1751, 1606–1491, and 1145. Mass spectra showed a quasi-molecular ion at *m/z* 247.0609. The molecular formula was therefore determined to be C_13_H_11_O_5_ (calculated [M + H]^+^ 247.0606).

^1^H-NMR (CDCl_3_) δ_H_ ppm: 8.12 (1H, d, *J* = 9.8 Hz, H-4), 7.62 (1H, d, *J* = 2.3 Hz, H-2′), 7.00 (1H, d, *J* = 2.3 Hz, H-3′), 6.29 (1H, d, *J* = 9.8 Hz, H-3), 4.16 (3H, s, 5-OCH_3_), 4.17 (3H, s, 8-OCH_3_). ^13^C-NMR (CDCl_3_) δ_C_ ppm: 160.6 (C-2), 150.0 (C-7), 145.2 (C-4), 144.4 (C-8), 143.8 (C-9), 139.5 (C-2′), 128.3 (C-5), 114.8 (C-6), 107.7 (C-10), 112.9 (C-3), 105.2 (C-3′), 61.8 (5-OCH_3_), 60.89 (8-OCH_3_). The NMR spectra data are consistent with published data [23].

#### Alloxanthoxyletin

The compound showed a UV maximum absorption (MeOH) at λ_max_ nm (log ε): 224 (4.14); 282 (4.11); and 324 (3.92). IR spectrum showed a band at ν_max_ (cm^− 1^): 3056; 2977; 2925; 2850; 1735; 1610–1568; and 1122. The mass spectrum showed a quasi-molecular ion [M + H]^+^ at *m/z* 259.0979. The molecular formula was determined to be C_15_H_15_O_4_ (calculated [M + H]^+^ 259.0970).

^1^H-NMR (CDCl_3_) δ_H_ ppm: 6.16 (1H, d, *J* = 9.6 Hz, H-3), 6.61 (1H, d, *J* = 9.9 Hz, H-4′), 7.96 (1H, d, *J* = 9.6 Hz, H-4), 5.5 (1H, d, *J* = 9.9 Hz, H-3′), 6.35 (1H, s, H-8), 3.87 (3H, s, 7-OCH_3_). ^13^C-NMR (CDCl_3_) δ_C_ ppm: 161.6 (C-2), 158.3 (C-7), 155.9 (C-9), 150.3 (C-5), 138.7 (C-4), 111.2 (C-3), 106.6 (C-6), 127.6 (C-3′), 116,1 (C-4′), 103.8 (C-10), 91.6 (C-8), 77.8 (C-2′), 28.0 (C-5′/6′), 56.0 (7-OCH_3_). The NMR spectra data are consistent with published data [24].

#### Methylevodionol

The compound showed a UV maximum absorption (MeOH) at λ_max_ nm (log ε): 312 (3.59); 287 (3.76); 257 (4.12) and 229 (4.23). IR spectrum showed a band at ν_max_ (cm^− 1^): 2974; 2925; 2850; 1622; 1598–1439 and 1126. The mass spectrum showed a quasi-molecular ion [M + H]^+^ at *m/z* 263.1288. The molecular formula was determined to be C_15_H_19_O_4_ (calculated [M + H]^+^ 263.1283).

^1^H-NMR (CDCl_3_) δ_H_ ppm: 5.51 (1H, d, *J* = 10.0 Hz, H-3), 6.46 (1H, d, *J* = 10.0 Hz, H-4), 6.18 (1H, s, H-9), 2.48 (3H, s, H-12), 1.41 (3H, s, H-13/14), 3.74 (3H, s, 6-OCH_3_), 3.76 (3H, s, 8-OCH_3_). ^13^C-NMR (CDCl_3_) δ_C_ ppm: 201.9 (C-11), 157.6 (C-8), 156.0 (C-10), 154.2 (C-6), 127.8 (C-3), 118.3 (C-7), 116.5 (C-4), 108.0 (C-5), 96.1 (C-9), 76.9 (C-2), 32.6 (C-12), 27.9 (C-13/14), 55.8 (6-OCH_3_), 55.8 (6-OCH_3_). The NMR spectra data are consistent with published data [22].

#### N-methylflindersine

The compound showed a UV maximum absorption (MeOH) at λ_max_ nm (log ε): 226 (4.29); 285 (3.20), 333 (3.65), 348 (3.69) and 365 (3.52) nm typical for a quinolinone skeleton [25]. IR spectrum showed a band at υ_max_ (cm^− 1^): 3083; 3055; 2974; 2896; 2879; 1730; 1612; 1452; 1236; and 1203. The mass spectrum showed a quasi-molecular ion [M + H]^+^ at *m/z* 242.1180. The molecular formula was determined to be C_15_H_16_NO_2_ (calculated [M + H]^+^ 242.1181).

^1^H-NMR (CDCl_3_) δ_H_ ppm: 5.52 (1H, d, *J* = 10.0 Hz, H-3), 6.74 (1H, d, *J* = 10.0 Hz, H-4), 7.30 (1H, d, H-7), 7.53 (1H, m, H-8), 7.95 (1H, dd, *J* = 7.8;1.5 Hz, H-10), 7.21 (1H, t, *J* = 7.8 Hz, H-9), 1.50 (3H, s, H-11/12), 3.68 (3H, s, N-CH_3_). ^13^C-NMR (CDCl_3_) δ_C_ ppm: 161.0 (C-5), 155.2 (C-10b), 130.9 (C-8), 139.3 (C-6a), 126.4 (C-3), 123.1 (C-10), 121.7 (C-9), 117.9 (C-4), 116.1 (C-10a), 114.1 (C-7), 105.8 (C-4a), 78.6 (C-2), 29.3 (N-CH_3_), 28.2 (C-11/12). The NMR spectra data are consistent with published data [26].

### Anti HCV activity and cytotoxicity of isolated compounds

The isolated compounds were evaluated against HCV in cultured Huh7it-1 cells in a dose dependent manner. The cytotoxicity was determined by MTT analysis. N-methylflindersine and O-methyloktadrenolon had stronger activities than the other tested-compounds. N-methylflindersine exhibited the strongest effect with an IC_50_ value of 3.8 ± 2.7 μg/ml, followed by moderate HCV inhibition activity of O-methyloktadrenolon and alloxanthoxyletin with IC_50_ values of 10.9 ± 1.2 μg/ml and 21.72 μg/ml, respectively. While, alloevodionol possess a moderate inhibition and the two remaining compounds, isopimpinellin, and methylevodionol did not show any inhibitive effect on HCV infection. The cellular viability assay demonstrated that all compounds had a negligible cytotoxicity effect with a CC_50_ > 90 μg/ml, except for O-methyloktadrenolon that had a CC_50_ of 63 ± 2.3 μg/ml (Table 1).

**Table 1:** The anti-HCV activity (IC_50_) and cytotoxicity (CC_50_) of compounds isolated from *M. latifolia* fruit extract

Sample	IC_**50**_ (μg/ml)	CC_**50**_ (μg/ml)	SI
O-methyloktadrenolon	10.9 ± 1.2	63 ± 2.3	5.8
Alloevodionol	41.1 ± 3.1	> 1000	> 24.3
Isopimpinellin	> 50	> 1000	> 20
Alloxanthoxyletin	21.72 ± 2.2	93 ± 3.0	4.3
Methylevodionol	> 50	310 ± 2.5	> 6.2
N-methylflindersine	3.8 ± 2.7	97 ± 3.3	25.5

Data represent mean ± SD of triplicate experiments

**Fig. 2:**
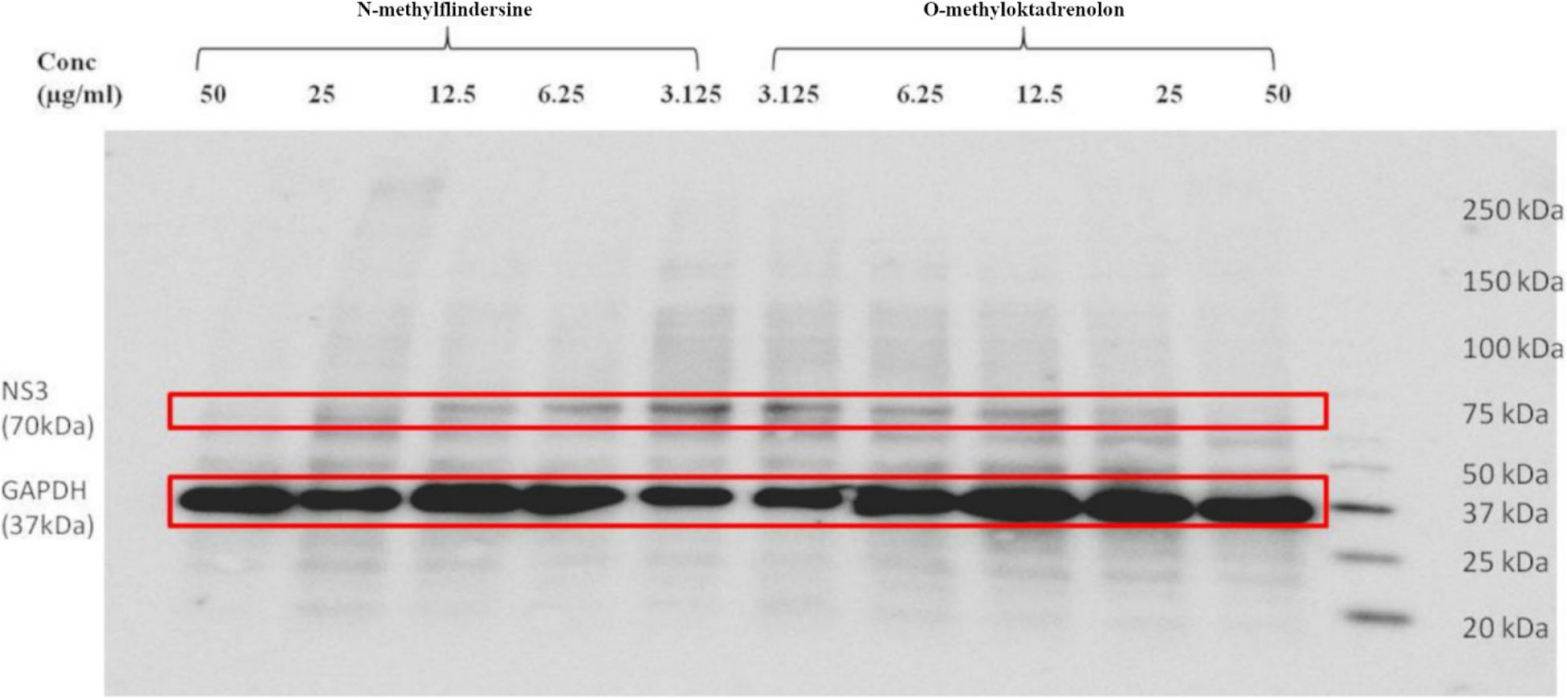
N-methylflindersin and O-methyloktadrenolon decreased HCV NS3 protein level. Culture of Huh7it-HCV infected cells (MOI = 0.5) was treated with various concentration of compounds. After 48 h of incubation, cells were subjected to western blot analysis using monoclonal antibody against HCV NS3 protein. GAPDH served as an internal control

Due to their efficacy, N-methylflindersine and O-methyloktadrenolon were tested on the different stages of HCV life cycle, in the entry step and post-entry steps. They were found to inhibit HCV in the post-entry step of HCV life cycle (Table 2). Western blot analysis was used to determine the mechanism by which this inhibition could occur and a reduction in the level of NS3 protease inhibitor was found. These results indicate that the compounds may interfere with the virus replication (Fig. 2).

**Table 2:** Mode of action of compounds isolated from *M. latifolia* fruit extract

Sample	Conc. μg/ml	% Inhibition			Mode of action
		During+Post inoculation	During inoculation	Post inoculation	
N-methylflindersine	25	83 ± 0.8	7.7 ± 1.8	84.1 ± 2.9	Post-entry inhibition
O-methyloktadrenolon	25	98.4 ± 0.4	9.1 ± 8.5	98 ± 0.9	Post-entry inhibition

Data represent mean ± SD of triplicate experiments

## Discussion

The six isolated compounds, O-methyloktadrenolon, alloevodionol, isopimpinellin, alloxanthoxyletin, methylevodionol, and N-methylflindersine were previously isolated from the other plants [27–31]. O-methyloktadrenolon, alloevodionol and methylevodionol have been isolated from *Melicope ptelefolia* leaves along with 15 other benzopyran compounds [30]. Isopimpinellin has been isolated from *Adiscanthus fusciflorus* (Rutaceae) and reported to possess an inhibitory activity against the enzyme adenine phosphoribosyltransferase (APRT) from *Leishmania* spp. [32]. This genus contains the causative agents of leishmaniasis. Isopimpinellin was also indicated to have chemo-preventive effects when administered orally after skin tumour initiation by 7,12-dimethylbenz[α]anthracene (DMBA) [33]. While, alloxanthoxyletin was previously isolated from *Pilocarpus goudotianus* (Rutaceae) [24]. An oil derived from the flower of *Melicope lunu-akenda* was found to contain ecodione (38.9%), (E)-β-ocimene (12.4%), isolycodolin (11.7%) and alloevadionol (10.6%) as major constituents. This oil exhibited antibacterial activity against Gram negative and Gram positive bacteria, in particular against *Salmonella typhi* and *Klebsiella pneumonia*, which are both human pathogens [34]. A quinolinone alkaloid, N-methylflindersine was previously isolated from stem bark of *Micromelum falcatum* and showed strong toxicity towards brine shrimp larvae with a LD_50_ value of 1.39 μg/ml [27]. It was also isolated from *Zanthoxylum integrifoliolum*, another species belonging to the Rutaceae family. N-methylflindersine has been found to inhibit N-formylmethionylleucylphenylalanine-induced superoxide production with an IC_50_ < 12 μM [28]. N-methylflindersine and methylevodionol have previously been isolated from the leaves of *Melicope denhamii*. In the same study, they showed moderate activity against murine leukemia P-388 cells with an IC_50_ value of 21.06 μg/ml and 11.98 μg/ml respectively [31]. In this study, anti-HCV activities of these compounds were evaluated and it was found that N-methylflindersine was revealed the strongest HCV inhibition activity with IC_50_ value of 3.8 ± 2.7 μg/ml, followed by O-methyloktadrenolon with an IC_50_ value of 10.9 ± 1.2 μg/ml. Alloevodionol and alloxanthoxyletin demonstrated a moderate activity with IC_50_ values of 41.1 ± 3.1 and 21.72 ± 2.2 μg/ml, respectively. On the other hand, methylevodionol and isopimpinellin, did not reveal anti-HCV activities at the highest concentration examined, 50 μg/ml (Table 1). Benzopyran compounds can form a benzoxazole moiety that can inhibit HCV by conjugation with a methylene thio (−SCH2-) linker that used to connect a heterobicycle with various aromatic rings by synthetically to form hybrid compounds for antiviral bioassays. The mode of action of the compound was found to be through inhibition in the post entry step by a reduction of HCV NS3 protein levels in dose dependent manner (Table 2; Fig. 2). N-methylflindersine is an alkaloid compound with the basic structure of quinoline. These quinolones compounds were known as antimicrobial, anticancer and antiallergic agents. Quinolones were reported to act as inhibitors of HCV NS5B RNA polymerase by binding to the allosteric site II (non-nucleoside inhibitor-site 2) of this protein [35–37]. Quinolone compounds consist of heterobicyclic aromatic compounds that may play an important role in anti-HCV activities.

## Conclusion

The alkaloid compound, N-methylflindersine which was isolated from the *M. latifolia* fruit mediated a strong anti-HCV activity through post-entry inhibition and HCV NS3 protein reduction. This result suggests that *M. latifolia* is a potential candidate for developing an anti-HCV agent.

## Data Availability

The all data used to support the findings of this study are available from the corresponding or the first authors upon request.

## Abbreviations

BSA: Bovine Serum Albumin
BCA: Bichincronic Acid
CC_50_: Cytotoxic Concentration 50%
DAAs: Direct Acting Anti-Virals
DAB: 3,3′-diaminobenzidine
DMEM: Dulbeco’s Modiffied Eagle Medium
DMSO: Dimethyl sulfoxide
FBS: Fetal Bovine Serum
GAPDH: Glyceraldehyde-3-phosphate dehydrogenase
HCV: Hepatitis C Virus
IC_50_: Inhibition Concentration 50%
IR: Infrared spectrophotometry
MOI: Multiple of Infection
NEAA: Non-Essential Amino Acids
NMR: Nuclear Magnetic Resonance
PBS: Phosphate Buffer Saline
SVR: Sustain virology respond
UV: Ultra violet
VLC: Vacuum Liquid Chromatography
